# The SPI-6 T6SS gene cluster from *Salmonella* Tennessee encodes a new antibacterial nuclease effector protein

**DOI:** 10.3389/fmicb.2026.1794835

**Published:** 2026-05-13

**Authors:** Carla Vargas-del Río, Jorge Urrea, Ayleen Parra-Calisto, Carlos J. Blondel, Andrea Avilés, Fernanda Salazar-Salas, Patricio Espinoza-Jara, Maira Mora, Dácil Rivera, Fernando A. Amaya, Carlos A. Santiviago, Andrea Moreno-Switt, David Pezoa

**Affiliations:** 1 Núcleo de Microbiología Traslacional para la Vigilancia e Innovación en Sistemas Sanitarios, Ambientales y Productivos MICRA. Facultad de Medicina Veterinaria y Agronomía, Universidad de Las Américas, Santiago, Chile; 2 Institute of Biomedical Sciences, Faculty of Medicine, Universidad Andres Bello, Santiago, Chile; 3 Laboratorio de Microbiología, Departamento de Bioquímica y Biología Molecular, Facultad de Ciencias Químicas y Farmacéuticas, Universidad de Chile, Santiago, Chile; 4 Escuela de Medicina, Facultad de Salud, Universidad del Alba, Santiago, Chile; 5 Escuela de Medicina Veterinaria, Facultad de Agronomía e Ingeniería Forestal, Facultad de Ciencias Biológicas y Facultad de Medicina, Pontificia Universidad Católica de Chile, Santiago, Chile

**Keywords:** effector, immunity protein, interbacterial competition, *Salmonella* Tennessee, T6SS

## Abstract

The type VI secretion system (T6SS) is a contact-dependent multiprotein apparatus widely distributed in Gram-negative bacteria that contributes to interbacterial competition and pathogenesis via a contractile mechanism. In *Salmonella*, five T6SS gene clusters have been identified within pathogenicity islands SPI-6, SPI-19, SPI-20, SPI-21 and SPI-22, which are differentially distributed among serotypes. One of the most studied and widely distributed T6SS corresponds to that encoded in SPI-6 (T6SS_SPI-6_), which contributes to *Salmonella* competition with the host microbiota and its interaction with infected host cells. Despite its relevance, there is still limited information available regarding the total number of effector proteins encoded within SPI-6 of different *Salmonella enterica* serotypes. In the present study, we characterized the SPI-6 T6SS gene cluster encoded in *Salmonella enterica* subspecies *enterica* serotype Tennessee (*S*. Tennessee), a pathogen frequently associated with foodborne gastrointestinal outbreaks. Interbacterial competition assays demonstrated that T6SS_SPI-6_ of *S.* Tennessee displays antibacterial activity. Additionally, we performed comparative genomic and bioinformatic analyses and identified an antibacterial Effector/Immunity protein (E/I) module encoding a putative effector with DNase activity (RhsA-HNHc) and its cognate immunity protein within the variable region 3 (VR3) of the SPI-6 T6SS gene cluster. Interbacterial competition assays confirmed the antibacterial activity of this novel E/I pair. In addition, heterologous expression assays showed that induction of the RhsA-HNHc effector led to significant *E. coli* growth inhibition, while co-expression with its putative immunity protein fully restored bacterial growth, thus demonstrating protection against toxicity. Finally, a nuclease activity assay demonstrated that RhsA-HNHc possesses DNase activity. Altogether, this study expands the experimentally validated SPI-6 T6SS effector repertoire beyond well-studied *Salmonella* serotypes, providing the first functional characterization of a DNase-type Rhs effector in *S*. Tennessee.

## Introduction

Type VI secretion system (T6SS) constitutes a significant fitness and virulence factor for a multitude of Gram-negative bacteria (Records, 2011; Basler, 2015; Cianfanelli et al., 2016; Navarro-Garcia et al., 2019). The T6SS is a molecular nanomachine comprised of three primary complexes: a contractile tail, a membrane complex, and a baseplate (Aschtgen et al., 2008; Brunet et al., 2015; Zoued et al., 2014; Durand et al., 2015; Logger et al., 2016; Nazarov et al., 2018; Rapisarda et al., 2019; Yin et al., 2019). The contractile tail is composed of an inner tube formed through the polymerization of the Hcp hexameric protein. At the tip of the Hcp tube, a needle-shaped VgrG protein trimer is assembled that interacts with the PAAR motif sharpening the tip of this structure (Brunet et al., 2015; Zoued et al., 2014; Ballister et al., 2008; Brunet et al., 2014; Douzi et al., 2014; Renault et al., 2018). The internal rigid Hcp tube is enveloped by a contractile sheath that is formed through the polymerization of TssB and TssC subunits (Durand et al., 2015; Leiman et al., 2009; Basler, 2015). Contraction of the sheath is responsible for providing the necessary energy for the injection of effector proteins into the target cell (Silverman et al., 2013). T6SS effectors are delivered as specialized effectors, fused to PAAR proteins and/or VgrG; or cargo effectors, in which the secretion is facilitated by non-covalent interactions with selected core components (Diniz and Coulthurst, 2015; Durand et al., 2014; Ma et al., 2017a; Pissaridou et al., 2018; Whitney et al., 2014). Most T6SS effectors are bacteria-specific, targeting the peptidoglycan (Ma and Mekalanos, 2010; Russell et al., 2012; Srikannathasan et al., 2013; Whitney et al., 2013; Berni et al., 2019; Wood et al., 2019) or the FtsZ protein involved in cell division (Ting et al., 2018). To prevent self-intoxication, these effectors are encoded together with their cognate immunity proteins in bicistronic units known as Effector/Immunity protein (E/I) modules (Russell et al., 2012). Some T6SS effectors are eukaryotic-specific and target molecules such as actin or microtubules (Ma and Mekalanos, 2010; Wood et al., 2019; Pukatzki et al., 2007; Miyata et al., 2011; Zheng et al., 2011; Durand et al., 2012; Lindgren et al., 2013; Schwarz et al., 2014; Heisler et al., 2015; Sana et al., 2015; Aubert et al., 2016; Jiang et al., 2016; Ray et al., 2017; Dutta et al., 2019; Tan et al., 2019), while others are able to attack both bacteria and eukaryotic cells (also known as trans-kingdom effectors), affecting conserved molecular targets such as NAD, nucleic acids and phospholipids, or through the formation of membrane pores (Whitney et al., 2015; Tang et al., 2018; Ahmad et al., 2019).

*Salmonella* is a pathogen that, once ingested, reaches the intestine and invades the epithelial cells, thereby stimulating inflammation (gastroenteritis), or crossing the intestinal barrier and disseminating throughout the reticuloendothelial system of the host (Haraga et al., 2008). *Salmonella* employs two distinct type III secretion systems (T3SSs) to deliver bacterial virulence proteins, known as effectors, directly into host cells. The T3SS encoded in *Salmonella* Pathogenicity Island 1 (SPI-1) is responsible for delivering effectors across the plasma membrane. This process is involved in the invasion of epithelial cells and the modulation of the inflammatory response. In contrast, the T3SS encoded in SPI-2 facilitates the delivery of effectors across the vacuolar membrane, thereby contributing to the survival and replication of intracellular *Salmonella*. In addition to the T3SSs, recent studies have elucidated the significance of T6SSs for *Salmonella* pathogenesis. *Salmonella* has been found to possess five T6SS gene clusters, which are encoded in pathogenicity islands SPI-6, SPI-19, SPI-20, SPI-21, and SPI-22. These gene clusters belong to different evolutionary lineages (Blondel et al., 2009; Fookes et al., 2011; Blondel et al., 2025). The most prevalent T6SS gene cluster is the one encoded in SPI-6, which is distributed in *Salmonella* isolated from various sources around the world (Wang et al., 2025; Amaya et al., 2024; Nicastro et al., 2026). Depending on the serotype, the SPI-6 T6SS gene cluster corresponds to a region of ~35–50 kb encoding ~30–45 open reading frames (ORFs), including each of the 13 T6SS core components (Blondel et al., 2009; Bao et al., 2019). The genetic architecture of the SPI-6 T6SS gene cluster exhibits a high degree of conservation among serotypes; however, structural variations are observed in three variable regions of the island (designated VR1, VR2, and VR3). VR1, located downstream of gene *tssC*, encodes the E/I pairs Tae2/Tai2 and Tae4/Tai4. Tae2 and Tae4 target the peptidoglycan having the ability to break the DD-crosslinks between D-mDAP and D-alanine, and the covalent bonds between D-Glu and mDAP of the tetrapeptide stem, respectively. Through this process, these T6SS effectors contribute to interbacterial competition and mice colonization by *Salmonella* (Russell et al., 2012; Sana et al., 2016). VR2 is located downstream of gene *tssM* and encodes numerous proteins of as yet unidentified function, in addition to two E/I pairs which possess peptidoglycan hydrolase activity. It has been hypothesized that Tge2/Tgi2P may possess N-acetylglucosaminidase activity (Whitney et al., 2013), while Tlde1/Tldi has been shown to exhibit L,D-carboxypeptidase activity against the peptide stems of the peptidoglycan layer (Sibinelli-Sousa et al., 2020; Lorente Cobo et al., 2022). Of note, most effector proteins identified in the *Salmonella* SPI-6 T6SS gene cluster are encoded within VR3 (Blondel et al., 2023). This region, located downstream of gene *tssI*, exhibits the greatest diversity of *Salmonella* T6SS effectors (Blondel et al., 2023). This phenomenon is attributed to the presence of a variable number of Rhs effector proteins that harbor C-terminal extensions carrying endonuclease domains, such as DNases and RNases, as well as deaminases and ADP-ribosyltransferases (Blondel et al., 2023; Amaya et al., 2024; Blondel et al., 2025; Nicastro et al., 2026).

Most of our knowledge on the presence and distribution of SPI-6 T6SS effector proteins derives from studies employing reference strains representing a limited number of serotypes such as *S.* and *S.* Typhimurium Dublin. Furthermore, only 9 of these effectors have been experimentally validated (Russell et al., 2012; Benz et al., 2013; Whitney et al., 2013; Koskiniemi et al., 2014; Sana et al., 2016; Sibinelli-Sousa et al., 2020; Amaya et al., 2022; Jurėnas et al., 2022; Lorente Cobo et al., 2022). This is a significant knowledge gap because the T6SS effector proteins are the ultimate mediators of T6SS activity. Consequently, their identification and characterization are crucial for a more comprehensive understanding of the *Salmonella* infectious cycle and its contribution to environmental fitness and pathogenic potential.

In this work, we identified a single SPI-6 T6SS gene cluster in *S*. Tennessee, a pathogen frequently associated with foodborne gastrointestinal outbreaks (Sheth et al., 2011), and demonstrated its contribution to interbacterial competition. Furthermore, our comparative genomic and bioinformatic analyses identified a new antibacterial E/I module within VR3 of the SPI-6 T6SS gene cluster encoding an effector protein with predicted nuclease activity (RhsA-HNHc). We confirmed the antibacterial activity of the predicted effector by interbacterial competition assays. In addition, heterologous expression of the predicted effector inhibited *E. coli* growth and co-expression with its putative immunity protein fully restored bacterial growth, thus demonstrating a protective effect against the toxicity exerted by the effector protein. Finally, via nuclease activity assays we confirmed that the predicted effector RhsA-HNHc exhibits DNase activity and that this activity is blocked in the presence of its cognate immunity.

## Results

### Characterization of the SPI-6 T6SS gene cluster in *S*. Tennessee FA1455

**Figure 1:**
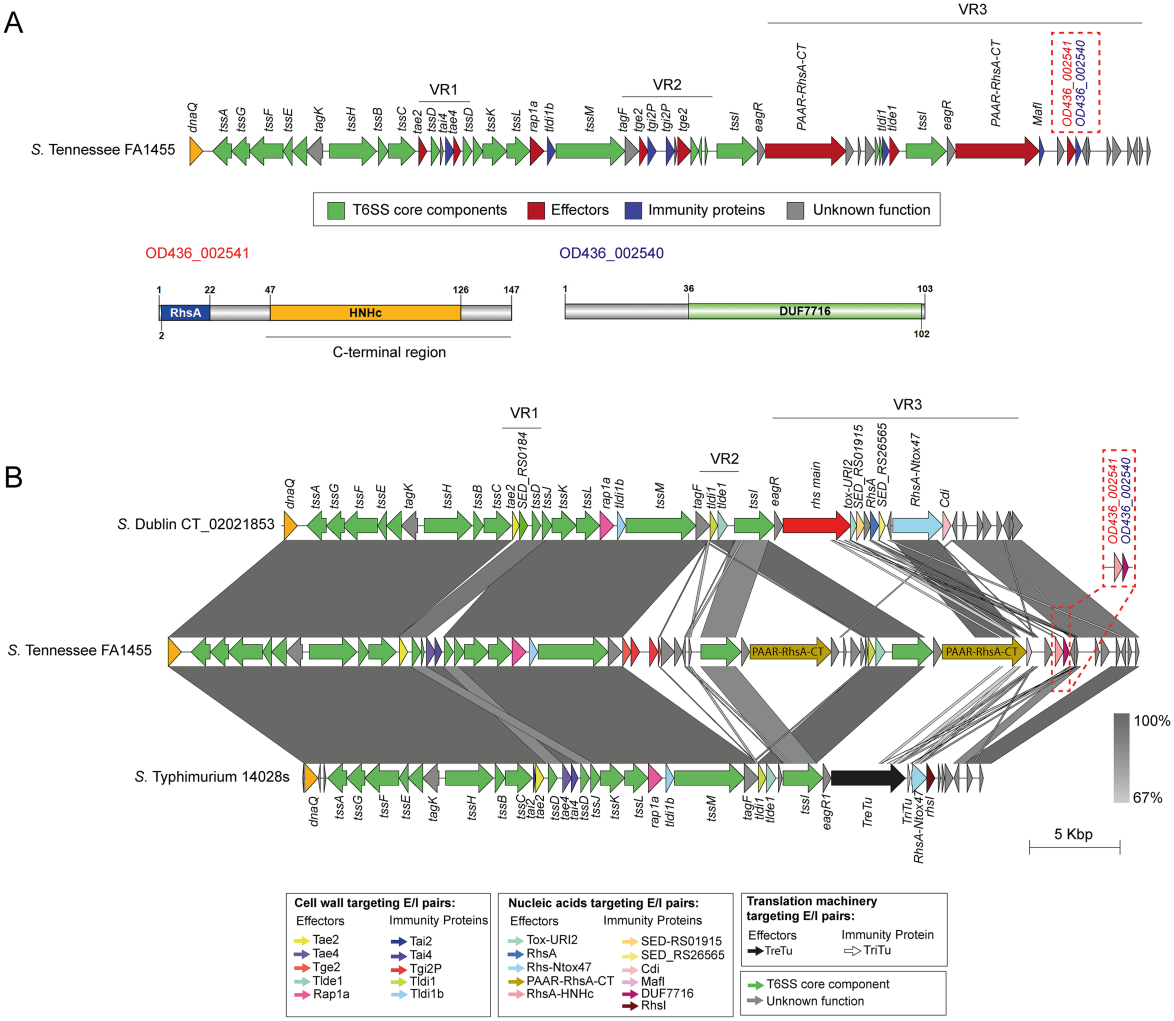
The SPI-6 T6SS gene cluster of *S.* Tennessee FA1455 encodes a new putative DNase effector. **(A)** Genomic organization of the SPI-6 T6SS cluster in *S.* Tennessee FA1455, including a schematic representation of the identified *OD436_002541*/*OD436_002540* E/I module. Genes predicted to encode effector and immunity proteins are shown in red and blue, respectively, whereas structural components of the T6SS are shown in green. **(B)** Comparative genomic analysis of the SPI-6 T6SS clusters from *S.* Tennessee FA1455, *S.* Dublin CT_02021853, and *S.* Typhimurium 14028s. Nucleotide sequence alignments were performed using BLASTn and visualized with EasyFig (Sullivan et al., 2011). ORFs in E/I modules are color-coded according to their experimentally validated or predicted functions. Grayscale shading represents the percentage of nucleotide sequence identity.

To identify T6SS gene clusters in *S*. Tennessee FA1455 (a multidrug-resistant strain isolated in Chilean surface waters in the study of Chen et al., 2024), we used the T6SS prediction tool from the SecreT6 web server^1^ and identified a single copy of the SPI-6 T6SS gene cluster in the genome of this isolate (Figure 1A).

The genetic organization of the SPI-6 T6SS gene cluster of *S*. Tennessee FA1455 revealed a similar genetic organization to the SPI-6 T6SS gene cluster of *S*. Dublin CT_02021853 and *S.* Typhimurium 14028s (Blondel et al., 2023), including the presence of the *tae2*/*tai2* E/I module in VR1 (Figure 1B). Nevertheless, the VR2 and VR3 regions of *S*. Tennessee FA1455 harbor additional ORFs, including two copies of the *tge2*/*tgi2P* E/I module and four ORFs of unknown function, in addition to the *tlde1*/*tldi1* E/I module present in the SPI-6 T6SS gene cluster of *S*. Dublin CT_02021853 and *S.* Typhimurium 14028s. Of note, VR3 in the SPI-6 T6SS gene cluster of *S*. Tennessee FA1455 harbors two *tssI-eagR-rhs* gene modules encoding two PAAR-RhsA T6SS effector proteins with C-terminal ends of unknown function (Table 1). One of these PAAR-RhsA effectors is encoded in a bicistronic unit with a gene encoding a protein with a MafI domain. This domain is frequently found in immunity proteins of bacterial toxins with RNase activity, strongly suggesting that this PAAR-Rhs effector may degrade RNA. It is interesting to note that these two *tssI-eagR-rhs* gene modules share no sequence identity with each other or with the corresponding module in *S.* Typhimurium 14028s and *S*. Dublin CT_02021853. This finding suggests that the *tssI-eagR-rhs* gene modules within the SPI-6 T6SS gene cluster of *S*. Tennessee FA1455 have a distinct evolutionary origin.

**Table 1:** Predicted T6SS effector and cognate immunity protein encoded in SPI-6 of *S*. Tennessee FA1455.

ORF	Prediction	Size (aa)	Predicted regions/domains (database, score and region in amino acids)
**OD436_002541**	Effector protein (Bastion6 score 0.84)	147	CDD: HNH/ENDO VII nuclease superfamily (score 4.46E-8, 47-126aa)
			BLASTp: HNH endonuclease MCL9480760.1 (score 7E-106, identity 100%)
			AF3/Foldseek: Similarity to ColE7 endonuclease *E. coli* (PDB 1 M08) (score 2.64E-11)
**OD436_002540**	Immunity protein of OD436_002541	103	Motif-finder: DUF7716 (score 3.2E-7, 36-102aa)
			Pfam: PF24832 DUF7716 (score 8.9E-13, 36-102aa)
			BLASTp: MULTISPECIES: DUF7716 domain-containing protein [*Salmonella*] WP_000849719.1 (score 1E-67, identity 100%)
OD436_002544	Effector protein (Bastion6 score 0.911)	1,560	CDD: proline-alanine–alanine-arginine (PAAR) domain, also containing C-terminal Rearrangement hotspot (Rhs) extensions PF05488 (score 6.40E-10, 266-342aa)
			CDD: RHS element core protein PF05593 (score 2.28E-111, 386-1445aa)
			BLASTp: RHS repeat-associated core domain-containing protein WP_000503222.1 (score 0.0, identity 100%)
OD436_002543	Immunity protein of OD436_002544	88	CDD: MafI family immunity protein IPR047880 (score 2.06E-23, 6-72aa)
			BLASTp: MafI family immunity protein WP_029412312.1 (score 4E-56, identity 100%)
OD436_002554	Effector protein (Bastion6 score 0.94)	1,499	CDD: proline-alanine–alanine-arginine (PAAR) domain, also containing C-terminal Rearrangement hotspot (Rhs) extensions PF05488 (score 5.06E-29, 188-262aa)
			CDD: RHS element core protein PF05593 (score 1.99E-45, 483-1378aa)
			BLASTp: PAAR domain-containing protein EJX3854495.1 (score 7E-56, identity 99.93%)
OD436_002553	Immunity Protein of OD436_002554	144	CDD: No domains identified
			Motif-finder: No domains identified
			AF3/Foldseek: Similarity to crystal structure of CDI complex *E. coli* (PDB 5T86) (score 4.02E-1)

The E/I pair experimentally validated in this study is highlighted in bold case.

### The SPI-6 of *S*. Tennessee FA1455 encodes a novel antibacterial T6SS candidate effector protein with nuclease activity

**Figure 2:**
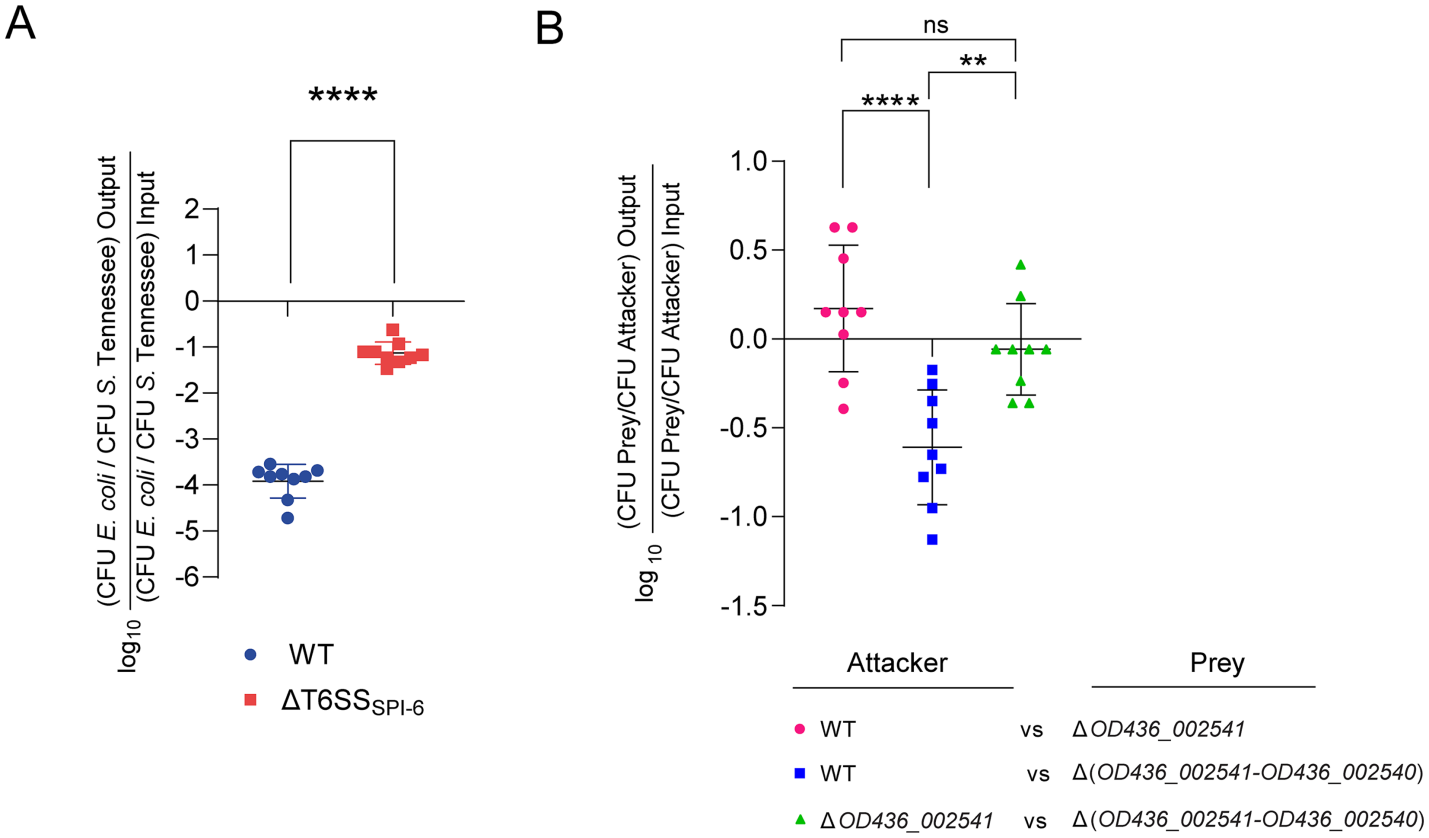
Contribution of the SPI-6 T6SS gene cluster and the *OD436_002541*/*OD436_002540* E/I module of *S.* Tennessee FA1455 to interbacterial competition. **(A)** *S.* Tennessee FA1455 (WT) or a ΔT6SS_SPI-6_ mutant were mixed with *E. coli* DH5α at a 1:1 attacker-to-prey ratio. Subsequently, 25 μL of the mixture was spotted in triplicate onto McConkey agar plates and incubated at 37 °C for 24 h. Following incubation, bacterial counts from each competition assay were determined by plating serial 10-fold dilutions onto LB agar supplemented with the appropriate antibiotics. *E. coli* DH5α was selected on Nal, whereas *S.* Tennessee strains were selected on Kan. Results are presented as the ratio of *E. coli* CFU (prey) to *S.* Tennessee CFU (attacker), normalized to the initial inoculum ratio and expressed as log_10_ values. Error bars indicate the standard error of the mean. Statistical significance was assessed using a two-tailed Student’s *t*-test (**** *p* < 0.0001). **(B)** *S.* Tennessee FA1455 (WT) and mutant derivatives lacking *OD436_002541* or the *OD436_002541*-*OD436_002540* module were mixed at a 1:1 attacker-to-prey ratio, and interbacterial competition assays were conducted as described in panel **(A)**. Statistical significance was assessed using a one-way ANOVA followed by Tukey’s multiple comparisons test (**p < 0.01; ****p < 0.0001; ns, not significant).

To determine the role of T6SS_SPI-6_ in interbacterial competition by *S*. Tennessee FA1455, a competitive assay was conducted on McConkey agar. Our results showed that the *E. coli* DH5α prey strain was significantly outcompeted after co-incubation with *S*. Tennessee FA1455. In contrast, the *S*. Tennessee FA1455 ΔT6SS_SPI-6_ strain showed a 1,000-fold lower ability to outcompete the *E. coli* DH5α prey strain in comparison with the wild-type strain (Figure 2A), indicating that T6SS_SPI-6_ provides *S*. Tennessee FA1455 with a competitive advantage over *E. coli* DH5α.

To gain insight into the T6SS_SPI-6_-dependent antibacterial activity of *S*. Tennessee FA1455, bioinformatic and comparative genomic analyses were performed to identify potential novel T6SS effector proteins and their cognate immunity proteins. To accomplish this, each ORF encoded within the SPI-6 T6SS gene cluster of *S*. Tennessee FA1455 was subjected to a comprehensive analysis, encompassing four distinct criteria. Firstly, an examination was conducted utilizing the Bastion6 prediction pipeline, a bioinformatics tool that employs amino acid sequence profiles, evolutionary information, and physicochemical properties to predict T6SS effectors. Secondly, a bioinformatic analysis was conducted to determine the presence of putative immunity proteins through the detection of signal peptides (SignalP), transmembrane domains (TMHMM), and operon prediction (Operon-mapper), as outlined by Taboada et al. (2018). Then, the following steps were taken to identify conserved domains and motifs linked to known T6SS effectors. First, the PROSITE, NCBI-CDD, Motif-Finder, and Pfam databases were utilized for identification. Second, functional predictions were made using the HHpred HMM-HMM prediction pipeline (Zimmermann et al., 2017) to perform HMM homology searches. Furthermore, an analysis of the SPI-6 T6SS gene cluster was conducted to identify potential unannotated ORFs that could encode putative effectors and cognate immunity proteins.

Interestingly, a putative effector protein was predicted to be encoded by *OD436_002541*, which is located downstream of the two *tssI-eagR-rhs* gene modules in VR3 (Table 1). This ORF encodes a 147 amino acid Rhs protein (Bastion6 score = 0.84) with a predicted HNHc domain (IPR028048) at its C-terminus (Figure 1A). In addition, *OD436_002541* is predicted to be a part of an operon with *OD436_002540* (Supplementary Figure 1), suggesting that *OD436_002541/OD436_002540* correspond to an E/I module.

To determine whether OD436_002541 contributes to the antibacterial activity mediated by T6SS_SPI-6_ in *S.* Tennessee, we generated isogenic mutant strains lacking either *OD436_002541* alone or the entire E/I module comprising *OD436_002541* and *OD436_002540*. The competitive fitness of these strains was assessed using bacterial competition assays on MacConkey agar. Deletion of the complete *OD436_002541/OD436_002540* module resulted in a significant reduction in survival following co-incubation with the wild-type strain (Figure 2B), consistent with the absence of the cognate immunity protein OD436_002540. In contrast, the mutant strain lacking *OD436_002541* and retaining *OD436_002540* did not display reduced survival when challenged with the wild-type strain (Figure 2B), indicating that the immunity protein is sufficient to protect against the toxic activity of the predicted effector OD436_002541 delivered by the attacker. As expected, the survival of the mutant lacking the entire *OD436_002541/OD436_002540* module was not compromised when co-incubated with an attacker strain lacking *OD436_002541* since the absence of the effector abolishes T6SS_SPI-6_-mediated killing, rendering the immunity protein dispensable (Figure 2B).

**Figure 3:**
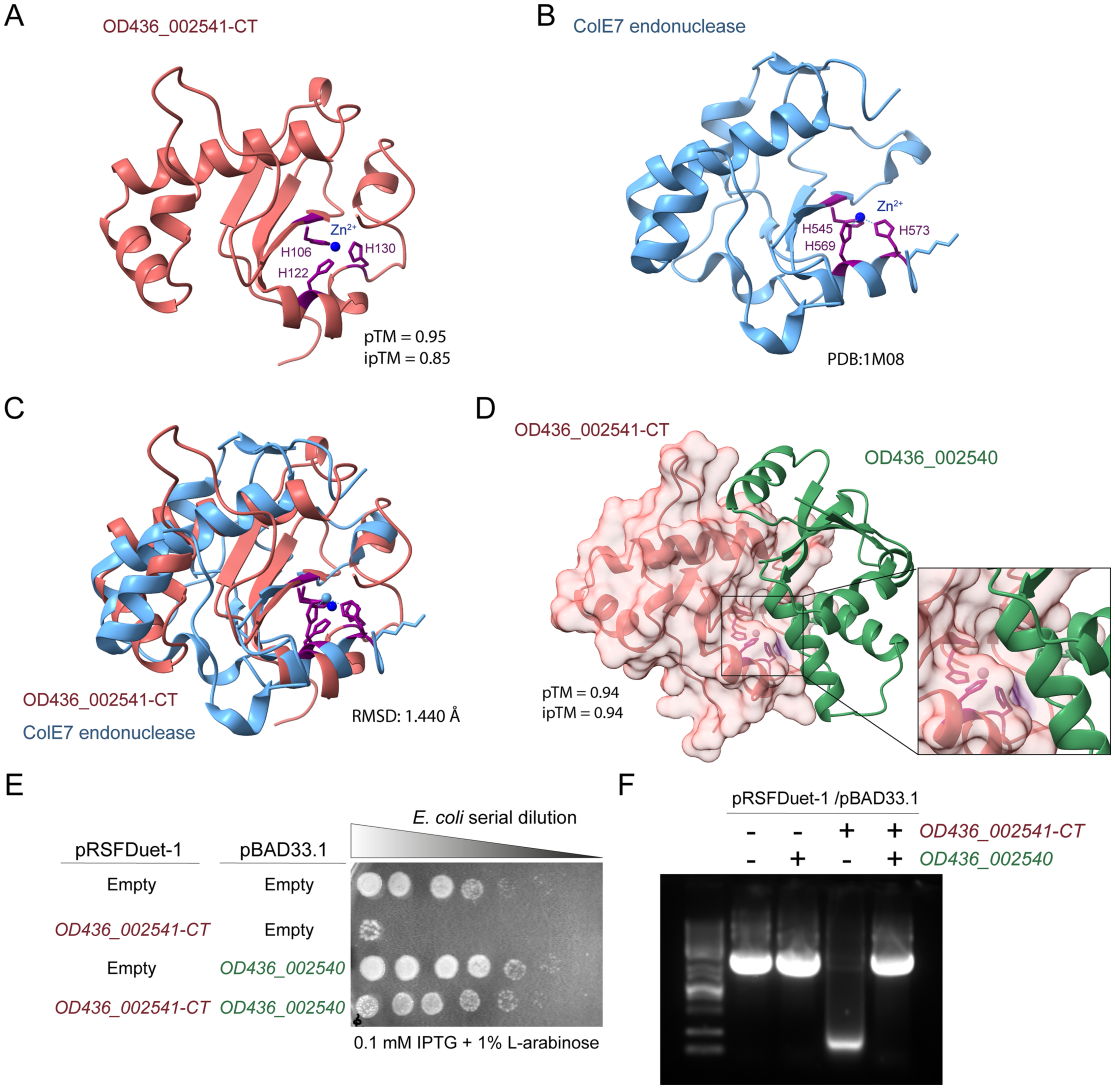
Structural and functional evidence supporting OD436_002541 as a DNase-type T6SS effector. **(A)** AlphaFold 3 model of OD436_002541-CT. **(B)** Crystal structure of ColE7 from *E. coli* K-12 strain W3110 (PDB 1M08). **(C)** Superimposition of the structures shown in panels **(A,B)**. **(D)** The predicted protein–protein complex structure of OD436_002541-CT/OD436_002540 T6SS E/I pair is shown with its corresponding ipTM score. The effector structure is shown in red and the predicted immunity protein (and its position relative to the putative T6SS effector) is shown in green. The histidine residues bound to Zn^2+^ are shown in purple, as predicted by AlphaFold 3. **(E)** Heterologous expression assays. The survival of *E. coli* BL21(DE3) derivatives carrying empty vector controls (pRSFDuet-1 and pBAD33.1) or plasmids encoding OD436_002541-CT and/or OD436_002540 was examined. Bacteria were serially diluted in LB broth (1:4) and 5 μL aliquots were dispensed onto LB agar plates supplemented with kanamycin and chloramphenicol, along with 0.1 mM IPTG and 1% L-arabinose to induce the synthesis of the effector and its putative immunity protein, respectively. Subsequently, bacteria were incubated at a temperature of 37 °C for 24 h. **(F)** *In vivo* plasmid DNA degradation assay was performed in *E. coli* BL21(DE3) derivatives carrying empty vector controls (pRSFDuet-1 and pBAD33.1) or plasmids encoding OD436_002541-CT and/or OD436_002540. The integrity of plasmids DNA was analyzed in 1% agarose gel after 3 h post induction with 0.1 mM IPTG and 1% L-arabinose.

Protein structure prediction analyses revealed that OD436_002541 includes the catalytic amino acid triad WHH, which is typically found in some members of the HNH/ENDO VII superfamily of nuclease proteins (Zhang et al., 2012). Foldseek analysis of the AlphaFold 3 predicted structure of the C-terminal domain of OD436_002541 revealed a high degree of similarity to the ColE7 endonuclease (PDB 1 M08) from the *E. coli* K-12 strain W3110 (Cheng et al., 2002) (Figures 3A,B). In both proteins, a Zn^2+^ ion is coordinated by three histidine residues (Figure 3). This Zn^2+^ ion is essential for the nuclease activity of members of the HNH/ENDO VII superfamily nucleases, including ColE7 (Cheng et al., 2002; Zhang et al., 2012). As illustrated in Figure 3C, this model showed a high degree of similarity between the predicted C-terminal domain of OD436_002541 and the catalytic domain of ColE7. This finding provides substantial evidence that the OD436_002541 protein harbors a DNase domain. As mentioned, our analysis indicate that *OD436_002541* is part of a bicistronic unit along with *OD436_002540* (Supplementary Figure 1). This latter ORF encodes a 103-amino-acid protein with a predicted DUF7716 domain, suggesting that OD436_002540 is the cognate immunity protein of OD436_002541. Indeed, an AlphaFold 3 analysis predicted that OD436_002541 and OD436_002540 constitute a T6SS E/I pair, with a high-confidence ipTM score of 0.93 (Figure 3D). Of note, the predicted interaction suggests that binding of the immunity protein sterically blocks the region of the effector protein including the 3 histidine residues coordinating the Zn^2+^ ion required for catalysis. As a control, the AlphaFold 3 analysis did not predict an interaction of OD436_002541 with a non-cognate immunity protein OD436_002543 (ipTM score of 0.35) (Supplementary Figure 2).

To gain insight into the mechanism of action of the predicted effector OD436_002541, heterologous toxicity assays were performed. The fundamental premise underlying these assays is that the effector protein should manifest toxicity when expressed in *E. coli*, and it is counteracted by the concomitant production of its cognate immunity protein. To evaluate if the expression of the HNHc domain of OD436_002541 is toxic to *E. coli* BL21(DE3), a DNA fragment encoding the C-terminal domain of OD436_002541 (OD436_002541-CT) was cloned into the expression vector pRSFDuet-1 under the control of the T7 promoter that can be induced by isopropyl β-D-1-thiogalactopyranoside (IPTG) (Table 2). We focused our analysis on the HNHc domain of OD436_002541, as antibacterial activity in this class of effectors is typically confined to the C-terminal region (Ma et al., 2017b), whereas the Rhs domain mainly serves as a secretion platform for the T6SS machinery (Donato et al., 2020). Furthermore, the DNA fragment that encodes the putative OD436_002540 immunity protein was cloned into the pBAD33.1 vector (Table 2). This construction allows expression of OD436_002540 under the control of the P_BAD_ promoter, which can be induced by L-arabinose. *E. coli* BL21(DE3) harboring empty control plasmids, or plasmids expressing OD436_002541-CT and/or OD436_002540, were serially diluted and plated on LB agar containing 0.1 mM IPTG and 1% L-arabinose. As shown in Figure 3E, expression of OD436_002541-CT was highly toxic to *E. coli*, while no toxicity was observed when only the OD436_002540 immunity protein was expressed. As expected, *E. coli* growth was not affected when the empty plasmids were induced. The toxicity of OD436_002541-CT was alleviated upon co-expression of the putative immunity protein OD436_002540 (Figure 3E).

**Table 2:** Bacterial strains and plasmids used in this study.

Strain or plasmid	Features	Source or reference
*Escherichia coli*		
DH5α	F^−^ Φ80∆*lacZ*(M15) ∆(*lacZYA-argF*) *U169* deoR *recA1 endA1 hsdR17*(r_k_^−^, m_k_^+^) *phoA supE44 thi-1 gyrA96 relA1 λ*^−^	Laboratory collection
BL21(DE3)	*E. coli B* F^−^ *ompT gal dcm lon hsdS_B_(r_B_^−^m_B_^−^) λ(DE3 [lacI lacUV5-T7p07 ind1 sam7 nin5]) [malB^+^]_K-12_(λ^S^)*	Laboratory collection
*Salmonella* Tennessee		
FA1455	Wild-type strain	Laboratory collection
ΔT6SS_SPI-6_	FA1455 Δ(*OD436_002532-OD436_002578*):: Kan	This study
Δ*OD436_002541*	FA1455 Δ*OD436_002541*:: Cam	This study
Δ(*OD436_002541*-*OD436_002540*)	FA1455 Δ(*OD436_002541*-*OD436_002540*):: Kan	This study
Plasmids		
pKD46	*bla P_BAD_ bet gam exo oriR101*(TS), Amp^R^	Datsenko and Wanner (2000)
pCLF2	Red-swap redesigned vector, Amp^R^, Cam^R^	GenBank HM047089
pCLF4	Red-swap redesigned vector, Amp^R^, Kan^R^	GenBank EU629214
pRSFDuet-1	*E. coli* expression vector. IPTG-inducible recombinant protein expression vector. The vector contains two multiple cloning sites (MCS1 and MCS2), each of which is preceded by a T7 promoter/*lac* operator and a ribosome binding site. Kan^R^. RSF replicon	Novagen
pBAD33.1	*E. coli* expression vector. Arabinose-inducible recombinant protein expression vector. Cm^R^. P15A replicon	NovoPro
pRSFDuet-1_OD436_002541-CT	pRSFDuet-1 derivative. Expressing the C-terminal domain of *OD436_002541* from *S.* Tennessee FA1455 fused to a His6 tag at the C-terminal end	This study
pBAD33.1_OD436_002540	pBAD33.1 derivative. Expressing the full-length *OD436_002540* from *S.* Tennessee FA1455 fused to a His6 tag at the C-terminal end	This study

The C-terminal HNHc domain of OD436_002541 is predicted to degrade DNA. To assess the DNase activity *in vivo* of OD436_002541-CT, we performed a plasmid DNA degradation assay in *E. coli*. To this end, *E. coli* cells harboring empty control plasmids (pRSFDuet-1 and pBAD33.1) or plasmids expressing OD436_002541-CT and/or OD436_002540, were induced with 0.1 mM IPTG and 1% L-arabinose in LB broth. Induction of OD436_002541-CT expression resulted in rapid degradation of the pRSFDuet-1 and pBAD33.1 (Figure 3F). Conversely, the expression of OD436_002540 in the absence of OD436_002541-CT expression showed no DNase activity, as observed when both empty vectors were induced (Figure 3F). In addition, co-expression of OD436_002541-CT with its putative immunity protein prevented plasmid DNA degradation, thus demonstrating protection against OD436_002541-CT toxicity. Together, our results indicate that OD436_002541/OD436_002540 is indeed an antibacterial T6SS E/I pair of *S*. Tennessee FA1455 with confirmed DNase activity.

## Discussion

The T6SS is a versatile machine that translocate a wide range of effector proteins into prokaryotic and/or eukaryotic cells. Therefore, it has evolved into a key molecular weapon in many bacterial pathogens. Five T6SS gene clusters have been identified in *Salmonella*, which are encoded within pathogenicity islands SPI-6, SPI-19, SPI-20, SPI-21, and SPI-22 (Blondel et al., 2009; Fookes et al., 2011). Although T6SS_SPI-6_ and T6SS_SPI-19_ have been associated with interbacterial competition and host colonization in some serotypes, there is still a lack of information concerning the global repertoire and distribution of effector proteins encoded within these gene clusters. The most prevalent T6SS gene cluster in *Salmonella* is encoded in SPI-6, in which an increasing number of candidate effector proteins have been identified in only a limited number of serotypes (Blondel et al., 2023; Amaya et al., 2024; Nicastro et al., 2026). Thus, the global *Salmonella* T6SS effector repertoire remains to be extensively identified and characterized in nearly all SPI-6 T6SS-encoding serotypes.

In this study, we identified the SPI-6 T6SS gene cluster in *S*. Tennessee, a serotype responsible for a number of outbreaks in the United States, many of which have been recently linked to consumption of contaminated peanut butter (Centers for Disease Control and Prevention (CDC), 2007; Sheth et al., 2011). By means of interbacterial competition assays, we showed that a ΔT6SS_SPI-6_ mutant of *S*. Tennessee FA1455 lost its ability to outcompete an *E. coli* DH5α prey strain in comparison with the wild-type strain. These results clearly indicate that T6SS_SPI-6_ contributes to the ability of *S*. Tennessee FA1455 to kill prey bacterial strains conferring a competitive advantage during interbacterial competition. Regarding the repertoire of effector proteins encoded within SPI-6 T6SS gene cluster, an increasing number of candidate T6SS effector proteins encoded within VR1, VR2 and VR3 have been identified in a limited number serotypes; however, only few of these effectors have been experimentally validated (Russell et al., 2012; Benz et al., 2013; Whitney et al., 2013; Koskiniemi et al., 2014; Sana et al., 2016; Sibinelli-Sousa et al., 2020; Amaya et al., 2022; Jurėnas et al., 2022; Lorente Cobo et al., 2022). This is a significant knowledge gap because the T6SS effector proteins are the ultimate mediators of T6SS activity and their deep characterization is fundamental to understanding their role in *Salmonella* pathogenesis and fitness. Interestingly, and as expected, we observed a great variability in the gene content of VR2 and, especially, VR3 of *S*. Tennessee FA1455. This is explained by the presence of two *tssI-eagR-rhs* gene modules in VR3 that harbors canonical T6SS PAAR-Rhs candidate effectors with C-terminal ends of unknown function. The association of one of these effectors with a gene encoding a MafI domain, a domain frequently found in immunity proteins of bacterial toxins with RNase activity (Zhang et al., 2012), strongly suggests that this Rhs effector may degrade RNA. Further experiments are required to confirm this hypothesis. In addition to these two *tssI-eagR-rhs* gene modules, we identified an antibacterial E/I pair including an effector protein with predicted nuclease activity (RhsA-HNHc) encoded within VR3 of *S*. Tennessee FA1455. By interbacterial competition assays we confirmed the antibacterial activity of OD436_002541 and the protective effect of its predicted cognate immunity protein, OD436_002540. Then, by heterologous expression assays we confirmed that expression of the C-terminal HNHc domain of OD436_002541 caused cell growth inhibition of *E. coli*. Furthermore, this toxic activity was neutralized by the co-expression of its cognate immunity protein OD436_002540. This contributes to our understanding of the versatility of the *Salmonella* T6SS effectors in targeting bacterial nucleic acids and underscores their status as a primary bacterial target of *Salmonella* T6SS effector proteins. In fact, the VR3 of the SPI-6 T6SS gene cluster encodes a wide variety of effector proteins carrying domains found in DNases, RNases, deaminases, and ADP-ribosyltransferases (Blondel et al., 2023; Amaya et al., 2024; Blondel et al., 2025; Nicastro et al., 2026). A notable observation is that most of these domains are fused to the C-terminal region of Rhs proteins, thereby contributing to the diversification of the molecular targets of T6SSs in *Salmonella*. Additionally, numerous Rhs proteins have been observed to possess C-terminal polymorphic endonuclease domains associated with T6SS effectors in *Salmonella* and other bacterial species (Zhang et al., 2012; Koskiniemi et al., 2014; Amaya et al., 2022). It has been established that Rhs proteins are characterized by the presence of YD-peptide repeats, which undergo a folding process that results in the formation of a substantial β-cage structure. This structural element has been observed to envelop and safeguard the C-terminal toxin domain, thereby enhancing the efficiency of T6SS secretion (Donato et al., 2020; Jurėnas et al., 2021; Günther et al., 2022). This observation lends credence to the hypothesis that the association of numerous T6SS effectors with these elements may be a result of selective pressures and recombination events.

An interesting finding is that OD436_002541 and OD436_002540 are predicted to establish a strong interaction using the AlphaFold 3 pipeline analysis (ipTM score of 0.93). In addition, the predicted effector OD436_002541 conserves the catalytic amino acid triad WHH found in a group of nuclease proteins belonging to the HNH/ENDO VII superfamily and has a high degree of structural homology to the ColE7 endonuclease from *E. coli* K-12 strain W3110. It is known that the bacterial toxin ColE7 degrades DNA non-specifically in target cells, leading to cell death (Cheng et al., 2002). A comparable ColE7 nuclease activity was identified for OD436_002541 through an *in vivo* plasmid degradation assay. This DNase activity was prevented by co-expression of the cognate immunity protein OD436_002540. Thus, we propose that OD436_002541/OD436_002540 is a functional T6SS E/I pair. However, additional experimental work is required to confirm that the effector protein OD436_002541 is secreted via T6SS_SPI-6_. In the same line, further experiments are required to confirm whether the genes comprising the E/I module *OD436_002541*/*OD436_002540* form a bicistronic operon. Such experiments may include RT-PCR spanning the intergenic region, co-expression analyses, and/or co-purification assays.

Altogether, our work broadens the repertoire of *Salmonella* T6SS effector proteins and provides evidence that the SPI-6 T6SS gene cluster of *S*. Tennessee contributes to interbacterial competition and harbors a great diversity of antibacterial effectors with nuclease activity. These effectors may provide *S*. Tennessee with a strong competitive advantage in complex microbial communities, such as surface waters or the inflamed gut.

## Materials and methods

### Identification of T6SS gene clusters in *S.* Tennessee FA1455

To identify T6SS gene clusters encoding each of the 13 core components of a T6SS in the genome of *S*. Tennesee FA1455 the T6SS prediction tool from the Secret6 web server (see text footnote 1) was utilized (Zhang et al., 2023). The selection of positive matches was determined by employing a BLASTp 2.10.1 + identity threshold for T6SS prediction greater than 30% and an E-value less than 0.0001. The utilization of these threshold values has yielded favorable outcomes in the identification of T6SS gene clusters within *Salmonella* genomes (Amaya et al., 2022; Blondel et al., 2023; Amaya et al., 2024). Each contig from the *S.* Tennessee strain FA1455 whole genome shotgun sequencing project (BioProject: PRJNA560080) was analyzed, identifying a complete T6SS gene cluster within contig ABIPKW010000006.1.

### Identification of candidate T6SS effectors

To identify putative T6SS effectors encoded within SPI-6 in the *S*. Tennessee FA1455 strain, each ORF was analyzed with the Bastion6 pipeline (Wang et al., 2018), with the exception of those encoding the 13 structural components of the T6SS. ORFs demonstrating a Bastion6 score of at least 0.6 were designated as potential T6SS effectors. It is noteworthy that a Bastion6 score of at least 0.5 is frequently employed as the default setting for the identification of T6SS effectors. However, in this study we chose a minimum score of 0.7 to conduct a more rigorous analysis. Each Bastion6 prediction was subjected to further analysis using tools implemented in the Operon-Mapper web server (Taboada et al., 2018) to determine its potential inclusion in a single transcriptional unit that also encoded a putative immunity protein. The term in question refers to a small protein with potential signal peptides (SignalP 6.0) and/or transmembrane domains (TMHMM 2.0). The identification of conserved functional domains and motifs in the candidate T6SS effectors was facilitated by the utilization of the PROSITE, NCBI-CDD, Motif-Finder, and Pfam databases (Kanehisa et al., 2002; Sigrist et al., 2013; Finn et al., 2014; Lu et al., 2019), which were integrated within the GenomeNet search engine. The E-value cutoff score was set at 0.01. In addition, biochemical functional predictions were conducted for the identified effector and immunity protein through HMM-based searches using the HHpred HMM-HMM comparison tool (Zimmermann et al., 2017). The new E/I pair comprised by the predicted effector OD436_002541 and its cognate immunity protein OD436_002540 corresponds to protein IDs EJX4025658.1 and EJX4025657.1, respectively.

### Bacterial strains and growth conditions

The bacterial strains used in this study are listed in Table 2. Bacteria were routinely grown in Luria-Bertani (LB) broth (10 g/L tryptone, 5 g/L yeast extract, 5 g/L NaCl) at 37 °C with continuous agitation at 180 rpm. LB broth was supplemented with ampicillin (Amp; 100 μg/mL), kanamycin (Kan; 50 μg/mL), chloramphenicol (Cam; 20 μg/mL), or nalidixic acid (Nal; 15 μg/mL), as needed. LB medium was solidified by the addition of agar (15 g/L). For interbacterial competition assays, bacteria were grown on McConkey agar plates (BD) at 37 °C for 24 h. For heterologous expression assays, bacteria were incubated at 37 °C for 24 h on LB agar plates supplemented with Kan and/or Cam and the corresponding inducers (i.e., 0.1 mM IPTG and/or 1% L-arabinose).

### Standard DNA techniques

Plasmid DNA was isolated using the “QIAprep Spin Miniprep Kit” (QIAGEN, MD, USA). PCR products were purified using the “QIAquick PCR Purification Kit” (QIAGEN, MD, USA). Analysis of DNA samples was performed by electrophoresis in 1% agarose gels and visualized under UV light after staining using GelRed (Biotium, CA, USA). Primers were designed using the SnapGene software^2^ and are listed in Table 3. PCR reaction mixes contained 1X buffer, 2 mM MgCl_2_, 100 nM dNTPs, 100 nM of each primer, 100 ng of template DNA and 0.5–1 U of Phusion High-Fidelity DNA Polymerase (NEB, USA). Standard conditions for amplification were: 1 min at 98 °C, followed by 30–35 cycles of 98 °C for 10 s, 55°–72° C (according to the appropriate Tm for each primer pair) for 30 s and 72 °C for a suitable time (15–30 s/kb), and a final extension step at 72 °C for 10 min. All amplifications were conducted in a “MultiGeneTC9600-G” thermal cycler (LabNet, NJ, USA).

**Table 3:** Primers used in this study.

Primer	Sequence^a^
Mutagenesis	
SPI-6_H1 + P1	AGGGTGTTTTTATACATCCTGTGAAGTAAAAAAAACCGTA*GTGTAGGCTGGAGCTGCTTC*
SPI-6_H2 + P2	GTGAACATGGCACATTAATTTGAAGCAGCTCTCATCCGGT*CATATGAATATCCTCCTTAG*
SPI-6_OUT5	GCTGGCTGCGCATGAATCGC
OD436_002541_H1 + P1	CATAGGGCTAATGGGCGGCCTGAATCTCTATGCTTATGCT*GTGTAGGCTGGAGCTGCTTC*
OD436_002541_H2 + P2	CTATATCCTCTTATCGACAACCTGTACCACCACCCCATTT*CATATGAATATCCTCCTTAG*
OD436_002540_H2 + P2	TACTTTAAGATTAGTCTAAAAAATCATCTTCTTCGAGATA*CATATGAATATCCTCCTTAG*
OD436_002541_Out5	GACGTTGTGATTTTACCGAT
K1	CAGTCATAGCCGAATAGCCT
C3	CAGCTGAACGGTCTGGTTATAGG
Cloning	
pRSFDuet_NdeI_4071_F	CACACATATGGGCGGCCTGAATCTCTATGC
pRSFDuet_XhoI_4071_R	CACACTCGAGTTAGTGGTGATGGTGATGATGTCGACAACCTGTACCACCAC
pBAD33.1_NdeI_4070_F	CACACATATGAAAACAATTGTAGGTTTTGA
pBAD33.1_HindIII_4070_R	CACAAAGCTTTTAGTGGTGATGGTGATGATGGTCTAAAAAATCATCTTCTT
DuetUP2 Primer	TTGTACACGGCCGCATAATC
T7 Terminator Primer	GCTAGTTATTGCTCAGCGG
pBAD Forward	ATGCCATAGCATTTTTATCC
pBAD Reverse	GATTTAATCTGTATCAGG

^a^Italics indicate the region that anneals to the 5′ or 3′ end of the antibiotic resistance cassette used for the mutagenesis.

### Construction of mutant strains

A mutant of *S.* Tennessee FA1455 with deletions of the whole SPI-6 T6SS gene cluster, the ORF of gene *OD436_002541* or the whole *OD436_002541*/*OD436_002540* E/I module were constructed by the one-step inactivation procedure using the Lambda Red recombination system (Datsenko and Wanner, 2000), with modifications (Santiviago et al., 2009). The oligonucleotides used for mutagenesis (Table 3) were made with 40 bases at the 5′ ends identical to the ends of the corresponding deletion, and 20 bases at the 3′ ends that anneal with the 5′ or 3′ end of a Cam or Kan resistance cassette flanked by Flp recombinase target (FRT) sites present in plasmids pCLF2 (GenBank accession number HM047089) and pCLF4 (GenBank accession number EU629214.1), respectively. pCLF2 and pCLF4 were employed as templates for the corresponding amplification of PCR products. *S.* Tennessee FA1455 was transformed with plasmid pKD46, which allows the inducible expression of the *λ* Red recombination system in the presence of L-arabinose. Then, bacteria carrying pKD46 were grown to an OD_600nm_ of 0.6 at 30 °C in LB broth supplemented with Amp and L-arabinose (10 mM). In the next step, bacteria were made electrocompetent through serial washes with ice-cold, sterile 15% glycerol, and transformed via electroporation with 500 to 600 ng of each PCR product. Transformants were selected at 37 °C on LB agar supplemented with the corresponding antibiotic. The correct insertion of the corresponding antibiotic resistance cassettes in each mutant was confirmed by PCR amplification using suitable primers (Table 3). To avoid any potential off-target effects of the chromosomal insertion of PCR products, we used generalized transduction to transfer the confirmed mutations to a *Salmonella* wild type background by means of the transducing phage P22 HT105/1 *int-201*.

### Interbacterial competition assays

Competition assays were performed to evaluate the ability of attacker strains to outcompete rival bacteria, following a previously described protocol (Ma et al., 2018) with minor modifications. In summary, the corresponding attacker and prey bacteria were cultivated overnight in LB broth at 37 °C. An aliquot (1 mL) of each culture was collected by centrifugation at 8,000 rpm for 2 min, after which the supernatant was discarded. Each bacterial pellet was washed thrice in sterile phosphate-buffered saline (PBS), adjusted to an OD_600nm_ of 0.5, and then mixed at a 1:1 attacker-to-prey ratio. Subsequently, aliquots (25 μL) of the mixture were spotted on McConkey agar plates in triplicate and incubated at 37 °C for a 24-h period. This condition has been documented as a factor that can induce the expression of T6SS gene clusters in *Salmonella* (Schroll et al., 2019; Amaya et al., 2022). Following the incubation period, the bacterial colonies were meticulously scraped from the McConkey agar plates and resuspended in 1 mL of sterile PBS. The CFU were then determined by plating serial dilutions on LB agar, which had been supplemented with the appropriate antibiotics. CFU obtained from interbacterial competition experiments were used for data analysis to calculate a competition index (CI). The CI values were calculated as a mean ratio of CFU of the prey to attacker strains normalized to the corresponding initial ratio, and converted logarithmically. The calculation of statistical significance was conducted using the GraphPad Prism 9.0 software, employing a two-tailed Student’s *t*-test or a one-way ANOVA followed by Tukey’s multiple comparisons test.

### Construction of plasmids for the heterologous expression of proteins in *E. coli*

For cloning the DNA sequence encoding OD436_002541 harboring a C-terminal His-Tag, a PCR amplicon was made using genomic DNA from *S.* Tennessee FA1455 and primers pRSFDuet_NdeI_4071_F and pRSFDuet_XhoI_4071_R (Table 3). Then, a purified PCR product digested with *Nde*I and *Xho*I was ligated into the multiple cloning site-2 (MCS2) of plasmid pRSFDuet-1 digested with the same enzymes to generate plasmid pRSFDuet-1_OD436_002541-CT (Table 2). The construction of the plasmid used for heterologous expression of the immunity protein OD436_002540 fused to a C-terminal His-Tag in *E. coli* involved the PCR amplification of the *OD436_002540* ORF. This was achieved using genomic DNA from *S*. Tennessee FA1455 and primers pBAD33.1_NdeI_4070_F and pBAD33.1_HindIII_4070_R (Table 3). The amplification product was then digested with *Nde*I and *Hind*III and cloned into pBAD33.1 previously digested with the same enzymes. This process resulted in the generation of plasmid pBAD33.1_OD436_002540 (Table 2).

### Heterologous toxicity assays

To evaluate the ability of OD436_002541 to inhibit bacterial growth and the protection given by its cognate immunity protein OD436_002540, overnight cultures of *E. coli* BL21(DE3) carrying recombinant plasmids pRSFDuet-1_OD436_002541-CT and pBAD33.1_OD436_002540 (Table 2) were serially diluted in LB broth (1,4). Then, aliquots (5 μL) were plated as spots onto LB agar plates supplemented with Kan and Cam, plus 0.1 mM IPTG and/or 1% L-arabinose to induce the expression of the effector and immunity protein, respectively. Following this, the plates were subjected to an incubation period at 37 °C for a duration of 24 h. Thereafter, the bacterial growth inhibition was determined by selected-plating assays. Derivatives of *E. coli* BL21(DE3) carrying both empty vectors (pRSFDuet-1 and pBAD33.1), as well as plasmid combinations pRSFDuet-1_4071/pBAD33.1 and pRSFDuet-1/pBAD33.1_OD436_002540, were used as controls.

The growth inhibition of OD436_002541 effector in *E. coli* BL21(DE3) was determined by growth on solid media in the presence of the inducer IPTG. Overnight cultures of *E. coli* BL21(DE3) cells with pRSFDuet-1 plasmid containing *OD436_002541*, *OD436_002540* or both genes were diluted 4-fold and aliquots (5 μL) were spotted onto LB agar plates containing Kan plus 0.1 mM IPTG and 1% L-arabinose to induce either the effector, immunity protein or both proteins, respectively. The plates were incubated at 37 °C for 24 h and the inhibition of bacterial growth was visually determined. *E. coli* BL21(DE3) containing empty pRSFDuet-1 was used as control.

### *In vivo* plasmid degradation analysis in *E. coli*

The *in vivo* plasmid degradation analysis in *E. coli* cells was conducted in accordance with the previously described procedures (Ma et al., 2014). Recombinant *E. coli* BL21(DE3) strains co-expressing OD436_002541 from plasmid pRSFDuet-1 and/or its putative immunity protein OD436_002540 from plasmid pBAD33.1 were inoculated in 5 mL of fresh LB broth with an initial OD_600nm_ of 0.01. IPTG and L-arabinose were added at a final concentration of 0.1 mM and 1%, respectively; coinciding with the moment at which the OD_600nm_ of these recombinant *E. coli* strains reached 0.2. The cells were harvested to obtain equal cell masses at 3 h post-IPTG induction. In the next step, plasmid DNA was extracted using the “QIAprep Spin Miniprep Kit” (QIAGEN, MD, USA), as described above. Then, plasmid DNA samples were analyzed by gel electrophoresis on a 1% agarose gel containing 1 × GelRed (Biotium, CA, USA) staining and run at 10 V/cm for 40 min. Finally, the DNA was subjected to visualization under UV light transillumination, using an UVITEC Alliance Q9 photodocumenter (UVITEC, Cambridge, UK).

### Protein structure prediction and homology modeling

Protein structure models of OD436_002541-CT and interaction between OD436_002541-CT and OD436_002540 were obtained by AlphaFold 3 (AlphaFold Server) (Abramson et al., 2024). The best model was used in downstream analyses (i.e., the rank_001 model). Protein structure visualization, template alignment, and superposition were performed using Pymol v3.1 and Matchmaker of UCSF ChimeraX (Meng et al., 2023). Protein structure searchers were performed with the Foldseek server (van Kempen et al., 2024).

## Data Availability

The original contributions presented in the study are included in the article/Supplementary material, further inquiries can be directed to the corresponding author.

## Footnotes

1
https://bioinfo-mml.sjtu.edu.cn/SecReT6/t6ss_prediction.php

2
www.snapgene.com

## Supplementary material

SUPPLEMENTARY FIGURE 1 Genetic organization of the predicted *OD436_002541*–*OD436_002540* operon. The operon structure and nucleotide sequence are presented, including the putative promoter elements (−35 and −10 regions) as predicted using Operon-mapper (Taboada et al., 2018). The associated probability score is also provided.

SUPPLEMENTARY FIGURE 2 Structural prediction of the OD436_002541-CT/OD436_002543 protein complex. The predicted protein–protein complex structure of OD436_002541-CT with a non-cognate immunity protein (OD436_002543) is shown with its corresponding ipTM score. The effector structure is shown in blue, while the non-cognate immunity protein (and its position relative to the putative T6SS effector) is shown in red, as predicted by AlphaFold 3.
