# Supplementary figures and images for: The SPI-6 T6SS gene cluster from Salmonella Tennessee encodes a new antibacterial nuclease effector protein
Source: Front Microbiol. 2026 May 13;17:1794835. doi: 10.3389/fmicb.2026.1794835 (PMC13224477; doi:10.3389/fmicb.2026.1794835)

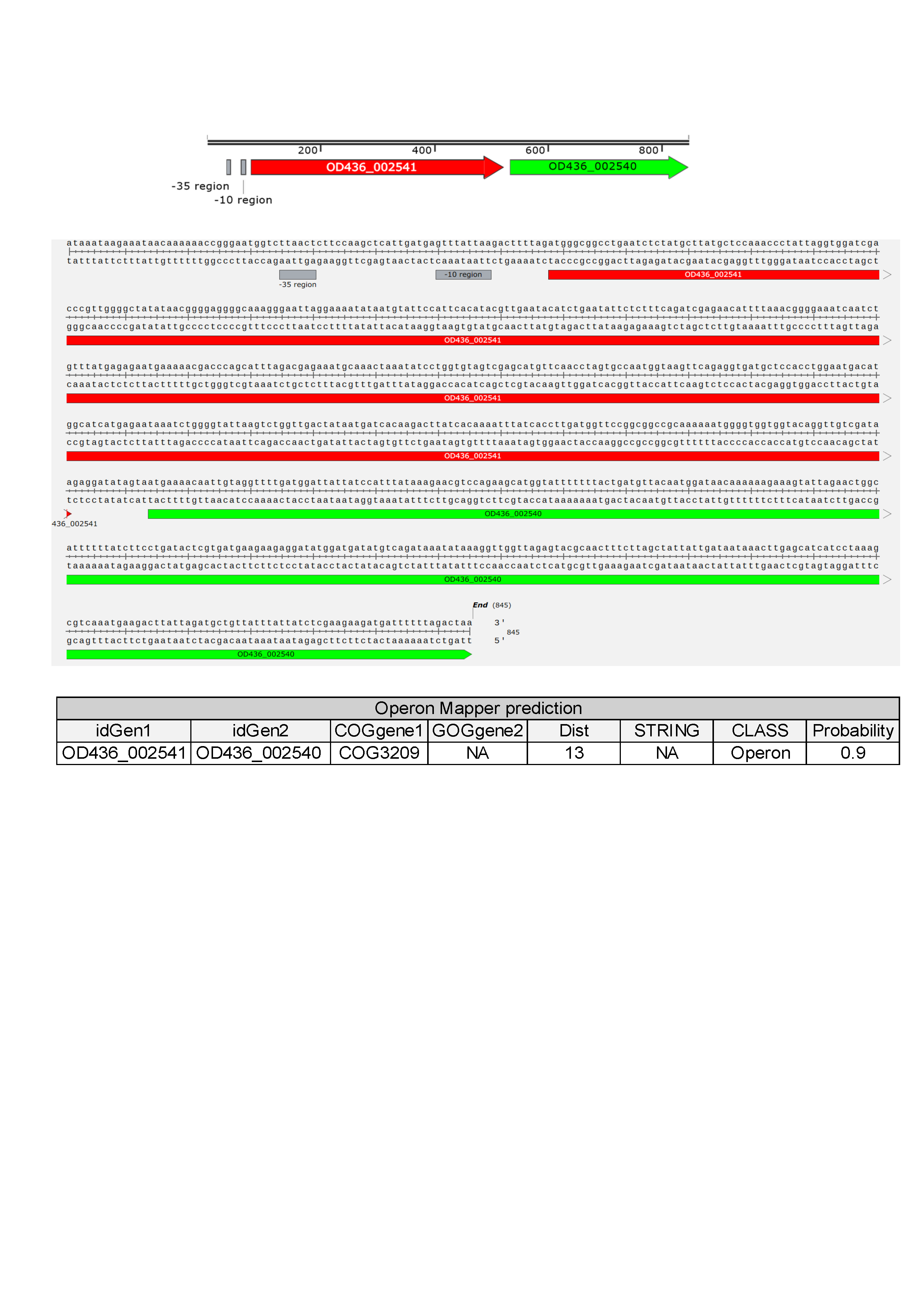

Supplement: SUPPLEMENTARY FIGURE 1 — Genetic organization of the predicted OD436_002541–OD436_002540 operon. The operon structure and nucleotide sequence are presented, including the putative promoter elements (−35 and −10 regions) as predicted using Operon-mapper (Taboada et al., 2018). The associated probability score is also provided. [file Image_1.TIF]

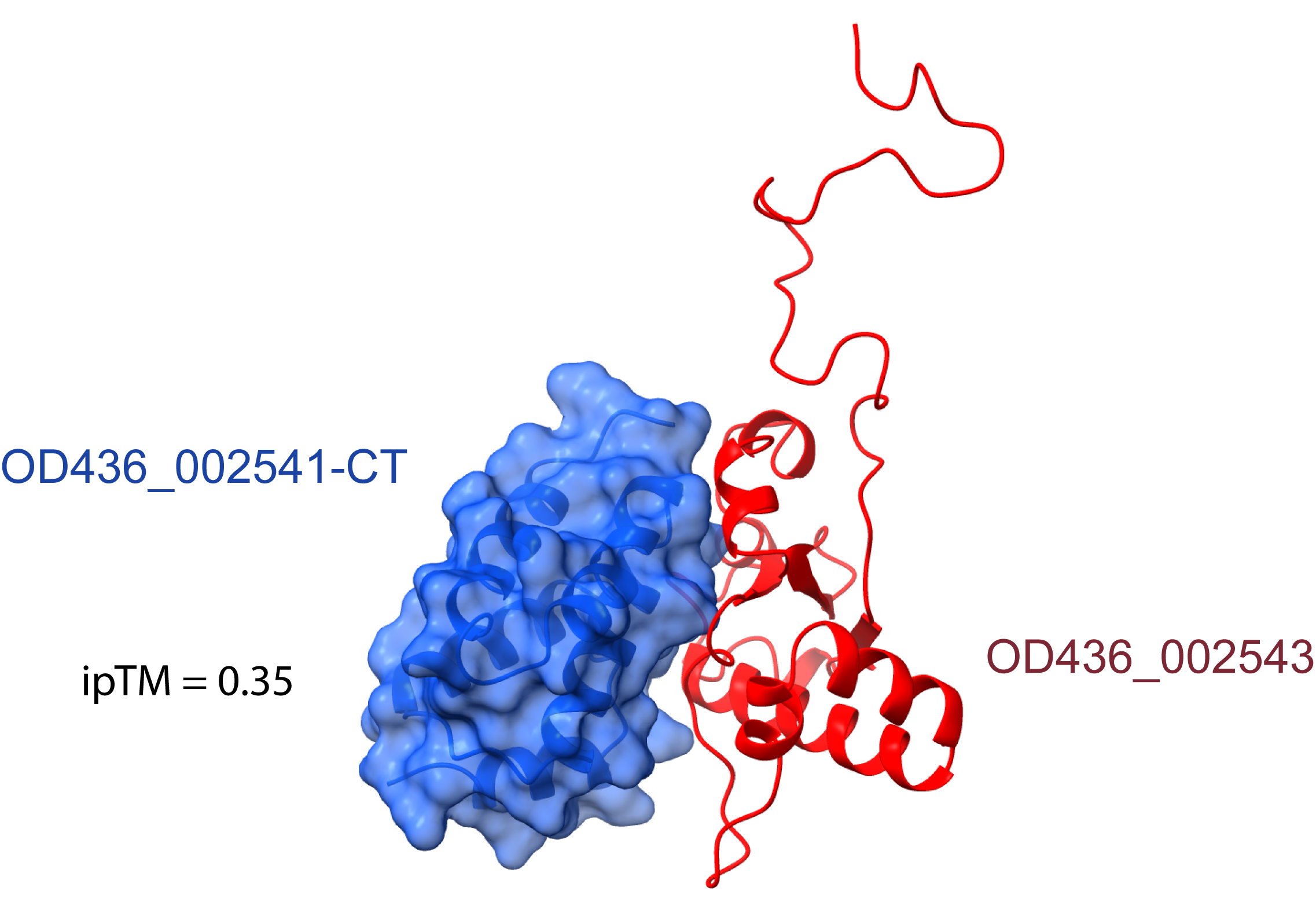

Supplement: SUPPLEMENTARY FIGURE 2 — Structural prediction of the OD436_002541-CT/OD436_002543 protein complex. The predicted protein–protein complex structure of OD436_002541-CT with a non-cognate immunity protein (OD436_002543) is shown with its corresponding ipTM score. The effector structure is shown in blue, while the non-cognate immunity protein (and its position relative to the putative T6SS effector) is shown in red, as predicted by AlphaFold 3. [file Image_2.TIF]
